# Supplementary material for: Super-Turing synaptic resistor circuits for intelligent morphing wing
Source: Commun Eng. 2025 Jun 16;4:109. doi: 10.1038/s44172-025-00437-y (PMC12170896; doi:10.1038/s44172-025-00437-y)
Supplement: Supplementary file 2 — Supplementary Information [file 44172_2025_437_MOESM2_ESM.pdf]

Supplementary Information

Super-Turing Synaptic Resistor Circuits for Intelligent Morphing Wing

Atharva Deo[1], Jungmin Lee[1], Dawei Gao[1], Rahul Shenoy[1], Kevin PT. Haughn[2], Zixuan Rong[1], Yong Hei[1], D. Qiao[1], Tanay Topac[3], Fu-Kuo Chang[3], Daniel J. Inman[2], and Yong Chen[1]*

[1]Departments of Mechanical and Aerospace Engineering, Electrical and Computer Engineering, Materials Science and Engineering, California NanoSystems Institute, University of California, Los Angeles, California 90095, USA

[2]Department of Aerospace Engineering, University of Michigan, Ann Arbor, MI 48104, USA

[3]Department of Aeronautics and Astronautics, Stanford University, Stanford, CA 94305, USA

[4]Department of Electrical and Computer Engineering, University of Massachusetts, Amherst, MA, USA

*Correspondence and requests for materials should be addressed to yongchen@seas.ucla.edu

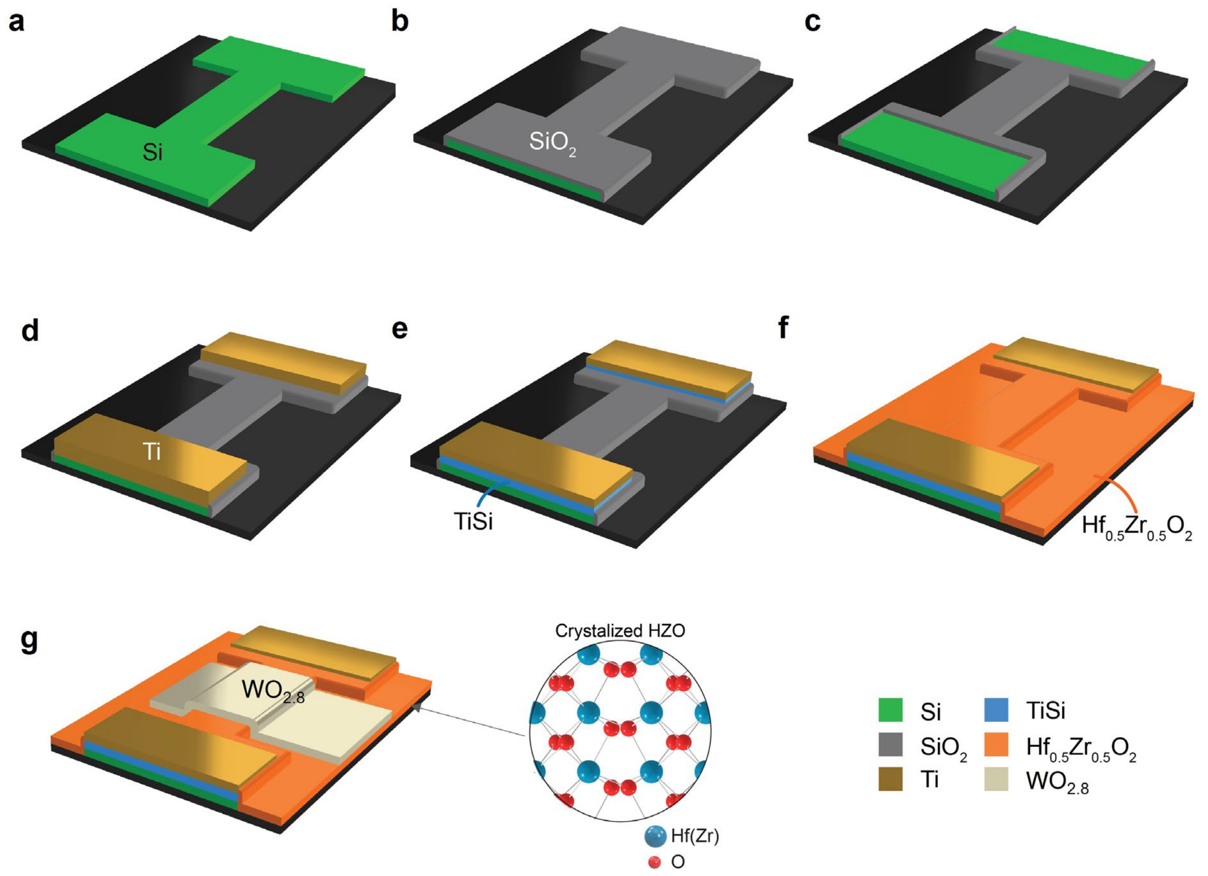

Fig. S1| The synstor circuit fabrication process. a, A Si channel is etched on a SiO_2 surface. **b**, The Si surface is oxidized and covered by a SiO_2 layer. **c**, The SiO_2 layer in contact areas is etched. **d**, Ti input and output electrodes are deposited in the contact areas, and **e**, are annealed to form a titanium silicide ($TiSi_{0.9}$) layer sandwiched between the Si channel and Ti input/output electrodes. **f**, A $Hf_{0.5}Zr_{0.5}O_2$ layer is deposited on the SiO_2 layer on the Si channel. **g**, A $WO_{2.8}$ reference electrode is made on the $Hf_{0.5}Zr_{0.5}O_2$ layer.

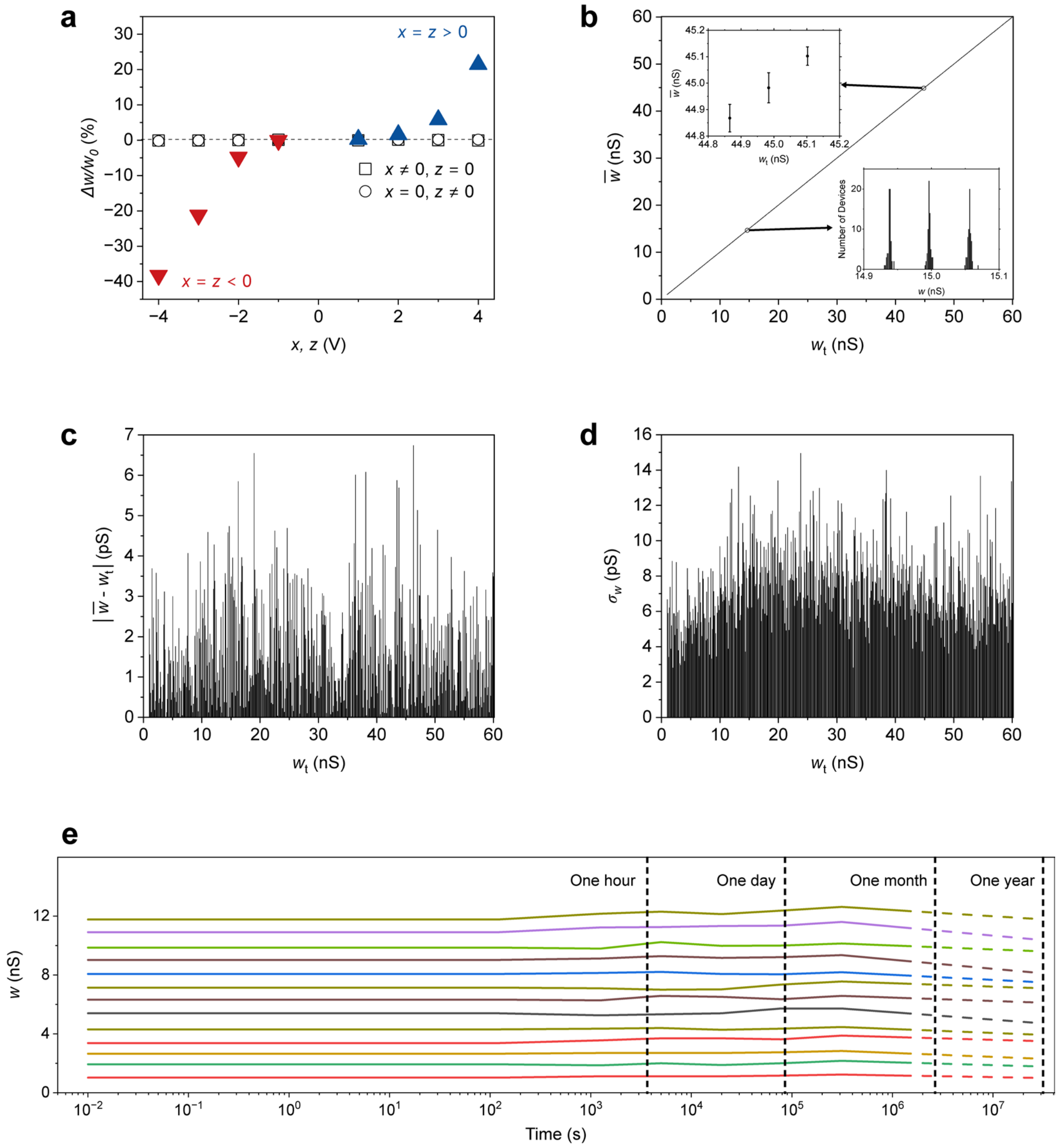

Fig. S2| The electric testing of synstor circuits. **a**, Conductance modification test: The percentage changes in the synstor conductance, $(\Delta w/w_0) \times 100\%$, are plotted against the amplitudes of 70 4 ms-wide x and z pulses applied on the input and output electrodes of the synstors under the various conditions of $-4\,V \leq x = z < 0$ (red triangles), $0 < x = z \leq 4\,V$ (blue triangles), $-4\,V \leq x \leq 4\,V$ and $z = 0$ (open squares), and $-4\,V \leq z \leq 4\,V$ and $x = 0$ (open circles). Circuit uniformity test: **b**, The analog learning accuracy was evaluated by fine-tuning 16 synstors in the circuit to precisely reach 500 distinct target conductance values, w_t, ranging from 1 nS to 60 nS and separated evenly by $\Delta w_t = 118\,pS$. The learning is achieved

by applying a train of paired $x_m = z_n$ pulses with a duration of $10\ \mu s$ and an amplitude of $-4\ V$ (or $4\ V$) to each synstor until their conductance values closely matched the targeted analog values. **c**, The absolute differences between the average analog conductance values $\overline{w}$ and the target analog conductance values of synstors w_t, $|\overline{w} - w_t|$, and **d,** the standard deviations of w, σ_w, are plotted against their corresponding target conductance values w_t. $|\overline{w} - w_t|$ values are smaller than $7.0\ pS$ or ~6 % of Δw_t, and σ_w values are below $15\ pS$ or ~13 % of Δw_t. **e**, Nonvolatile conductance retention test: After 13 synstors were adjusted to 13 evenly spaced analog conductance levels, their conductance was monitored over $10^6\ s$ (solid lines) at room temperature and projected for one year (dashed lines). Although some shifts in conductance were observed, the projected conductance levels remain distinct without overlap. The retention of these analog conductance levels is projected to last for more than a year.

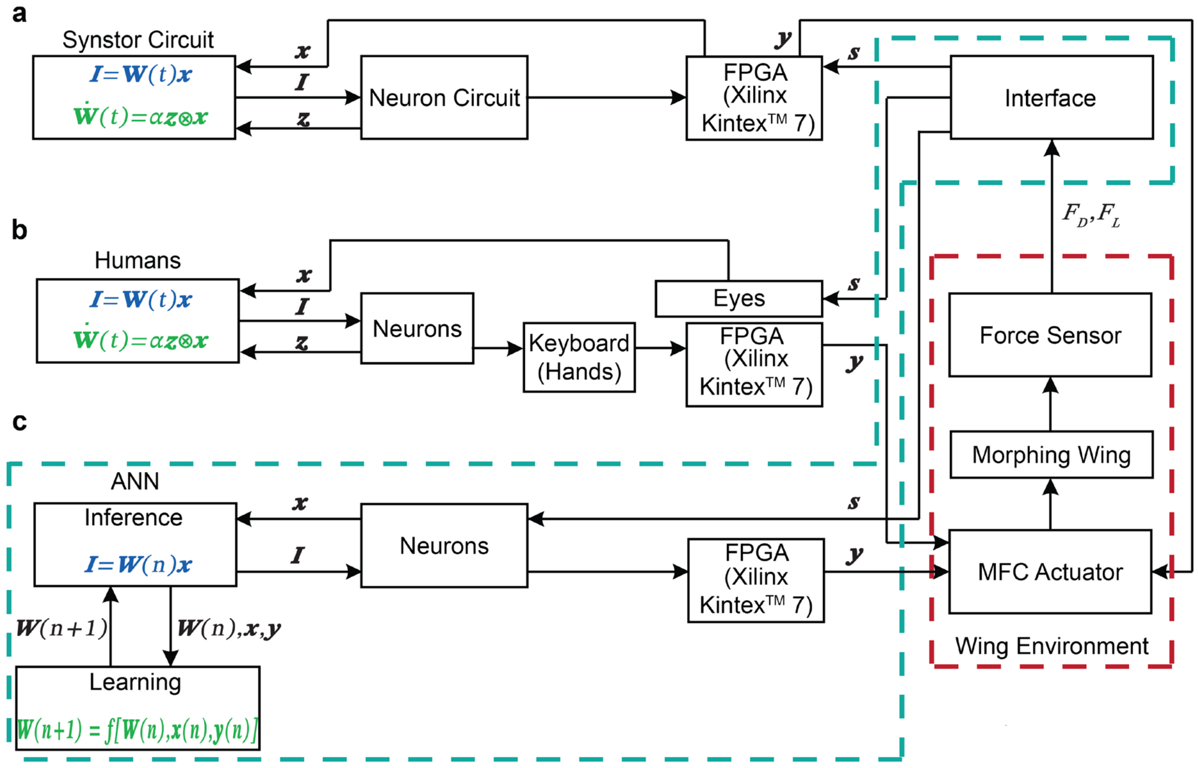

Fig. S3| Schematics of experimental systems. Experimental systems used to control a spaceship by **a,** a synstor circuit, **b,** human neurobiological circuits with concurrent execution of inference ($\boldsymbol{I} = \boldsymbol{W}(t)\ \boldsymbol{x}$) and learning ($\dot{\boldsymbol{W}}(t) = \alpha\ \boldsymbol{z} \otimes \boldsymbol{x}$) algorithms, and **c,** a computer-based ANN, sequentially executing inference $\boldsymbol{I} = \boldsymbol{W}(n)\boldsymbol{x}$ and learning $\boldsymbol{W}(n+1) = f[\boldsymbol{W}(n), \boldsymbol{x}(n), \boldsymbol{y}(n)]$ algorithms.

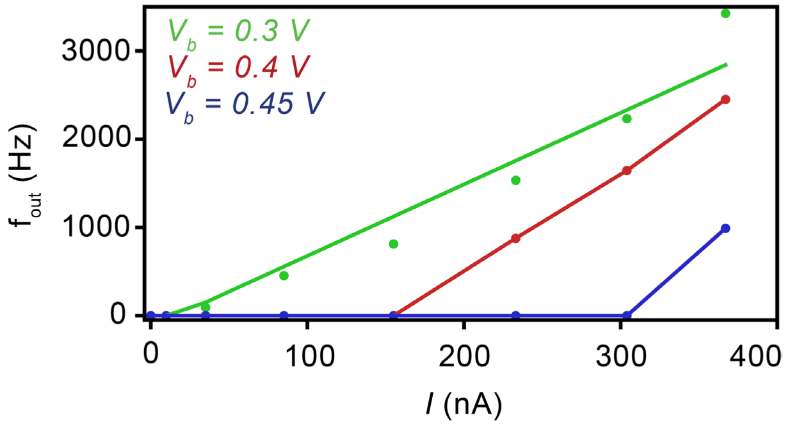

Fig. S4| The electric properties of neuron circuits. The frequency, f_{out}, of the y voltage pulses output from a neuron circuit is plotted against the current I input to the neuron circuit, with the control voltage (V_b) for the leakage current set to 0.3 V (green dots), 0.4 V (red dots), and 0.45 V (blue dots), respectively. As shown by the solid lines, f_{out} can be best-fitted by a liner threshold function $f_{out} = \begin{cases} 0 & when\ I < I_{th} \\ k_b I & when\ I \geq I_{th} \end{cases}$ with I_{th} and k_b as the fitting parameters.

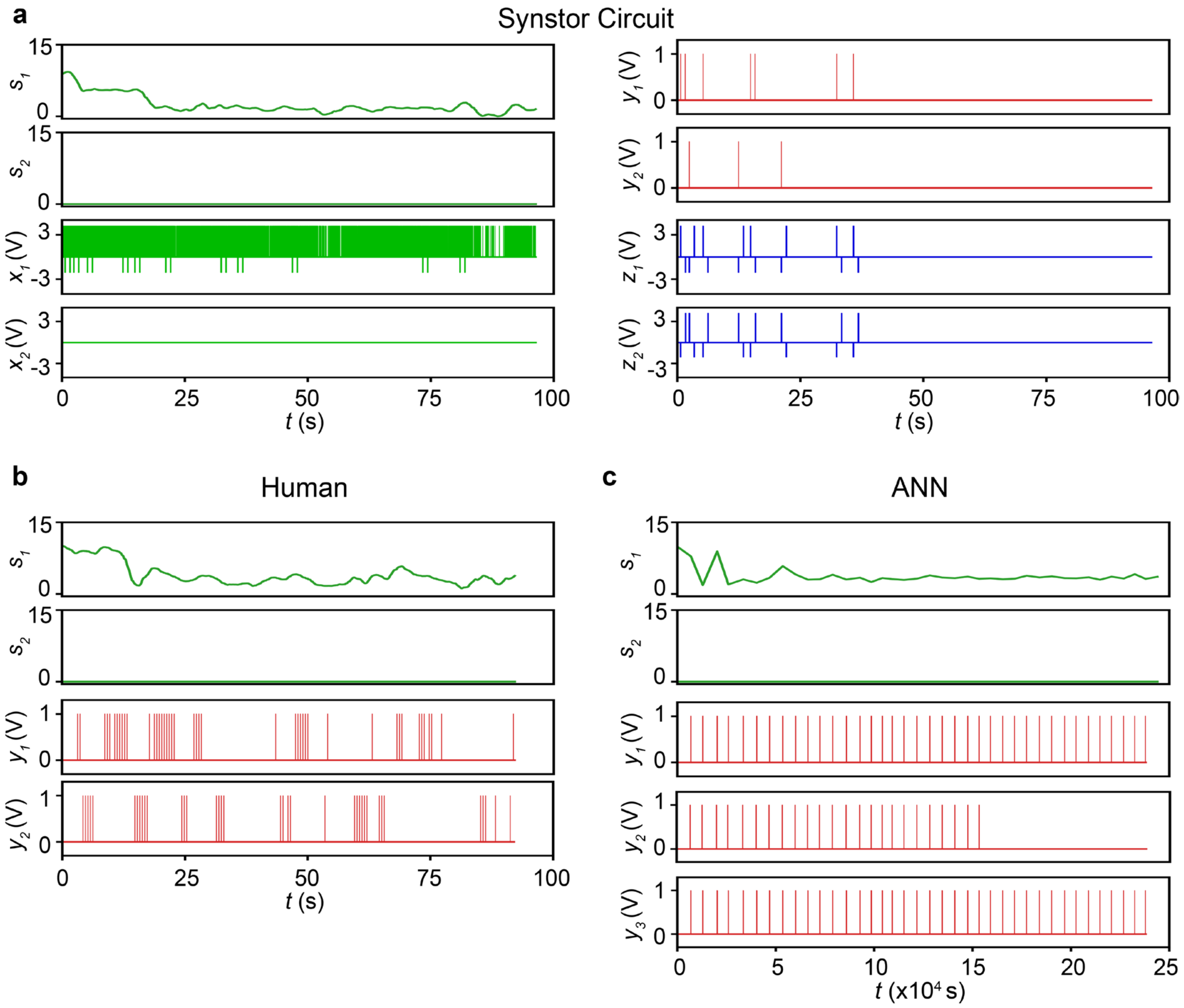

Fig. S5| Signals from experiments with a morphing wing controlled by a synstor circuit, a human operator, and an ANN in pre-stall conditions. The sensing signals, $\boldsymbol{s}$, including the drag-to-lift force ratio (s_1) and the magnitude of the fluctuation in the drag-to-lift force ratio (s_2), the input voltage pulses, x_1 and x_2, converted from the s_1 and s_2 signals, the output voltage pulses from neuron circuits, y_1 and y_2, used to modify the wing shape, and the voltage pulses (z_1 and z_2) applied on the output electrodes are displayed against time t in experiments where the morphing wing was controlled by **a,** a synstor circuit, **b,** a human operator, and **c,** and ANN running on a computer.

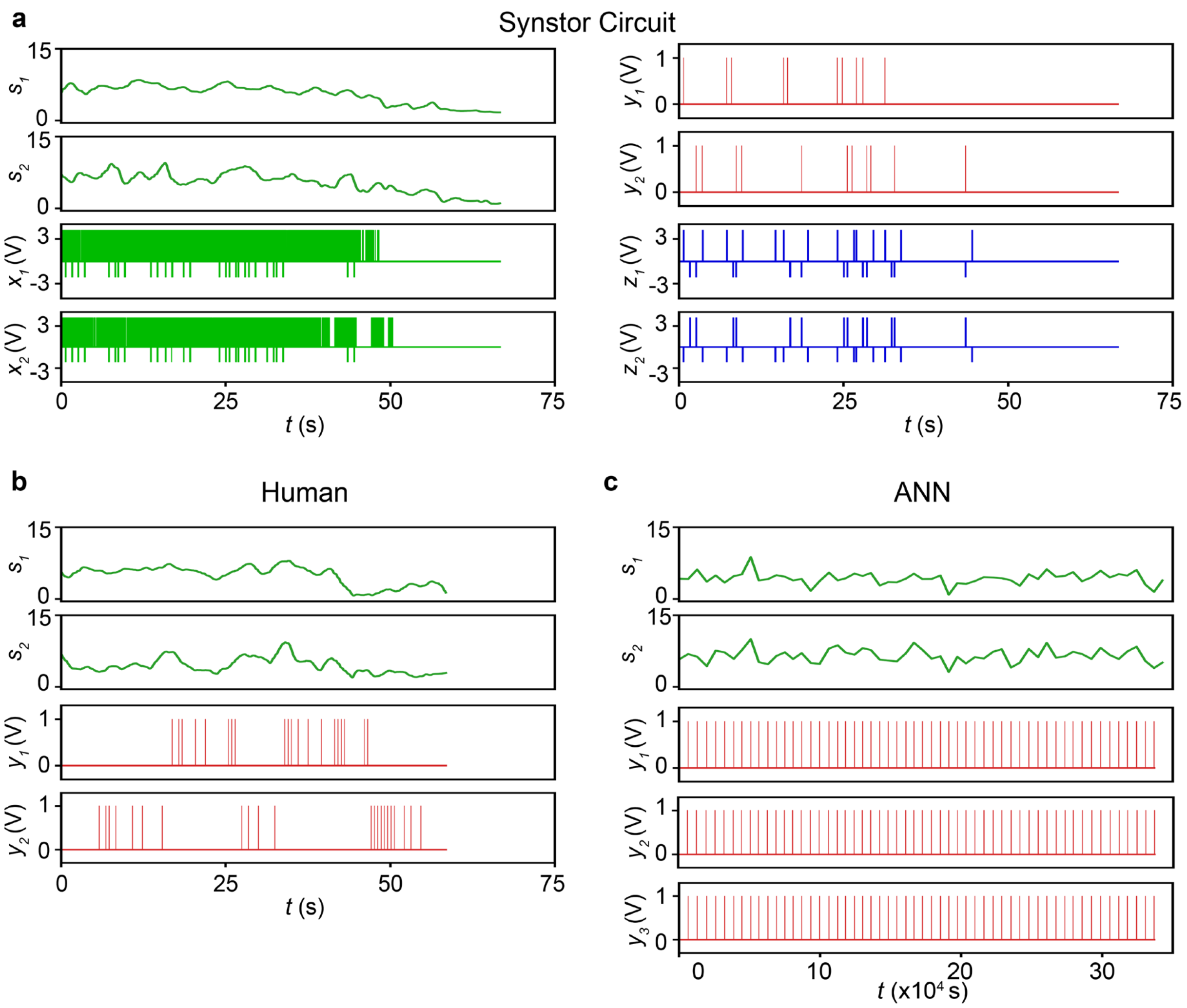

Fig. S6| Signals from experiments with a morphing wing controlled by a synstor circuit, a human operator, and an ANN in stall conditions. The sensing signals, $\boldsymbol{s}$, including the drag-to-lift force ratio (s_1) and the magnitude of the fluctuation in the drag-to-lift force ratio (s_2), the input voltage pulses, x_1 and x_2, converted from the s_1 and s_2 signals, the output voltage pulses from neuron circuits, y_1 and y_2, used to modify the wing shape, and the voltage pulses (z_1 and z_2) applied on the output electrodes are displayed against time t in experiments where the morphing wing was controlled by **a,** a synstor circuit, **b,** a human operator, and **c,** and ANN running on a computer.

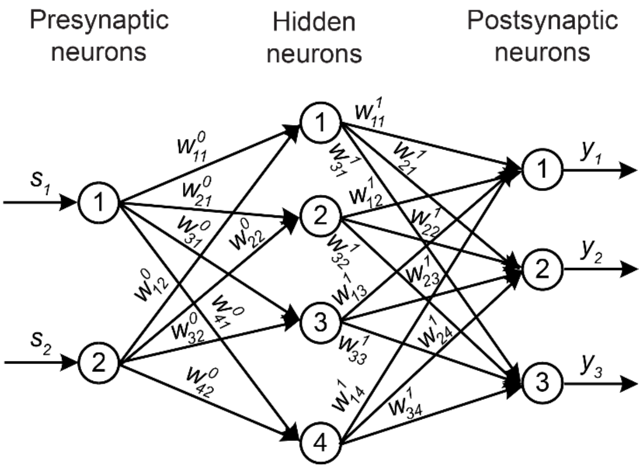

Fig. S7| Schematic of the ANN structure for controlling the morphing wing. A schematic illustrates the structure of an ANN used to control the morphing wing. The ANN consists of an input layer with 2 neurons, a hidden layer with 4 neurons, and an output layer with 3 neurons. The synaptic weights (***W***) are labeled on the lines representing the artificial synapses connected the neurons.

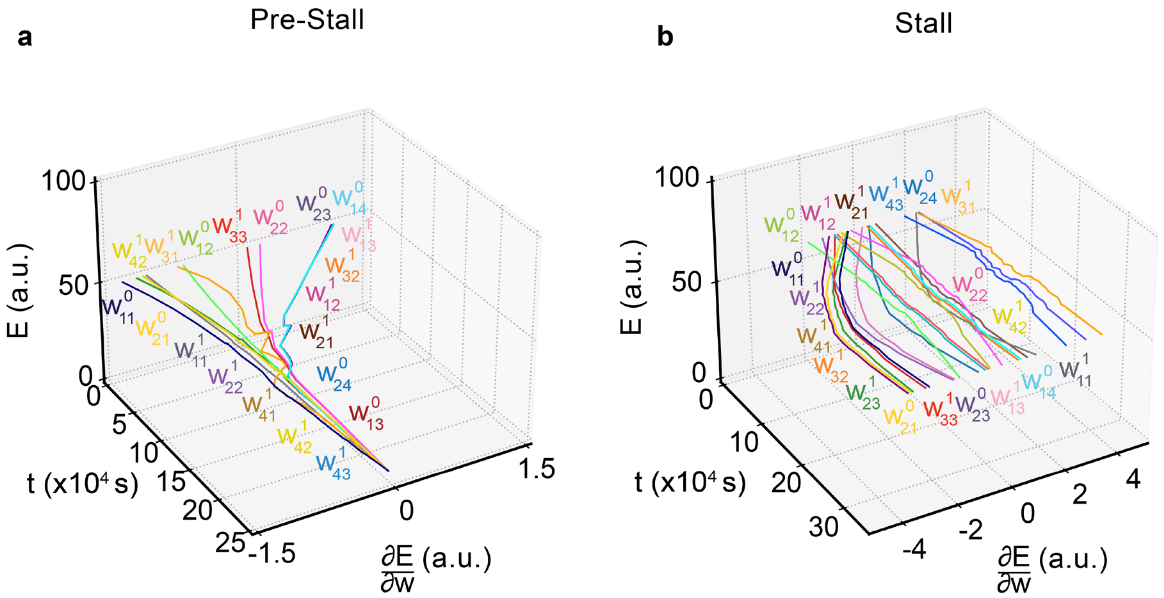

Fig. S8| Analysis of experiment results for the ANN controlled wing in pre-stall and stall conditions. E and $\frac{\partial E}{\partial w_{nm}}$ (in arbitrary unit) for the wing controlled by the ANN are plotted against time t in experiments for **a,** the wing in the pre-stall condition with an 8° angle of attack, and **b,** the wing in the stall condition with an 18° angle of attack.

Movie S1 caption

Movie S1. Experiments of morphing wing controlled by a synstor circuit, a human operator, and an ANN in pre-stall condition with an 8° angle of attack. A morphing wing in a wing tunnel is controlled by **a**, a synstor circuit, **b**, a human operator, and **c**, an ANN running in a computer. (Top) The videos show that the wing in a wing tunnel. (Bottom) Objective functions, $E = \frac{1}{2}\boldsymbol{s}^2$, with s_1 as the drag-to-lift force ratio and s_2 as the magnitude of the fluctuation of the drag-to-lift force ratio, is plotted against time t.

Movie S2 caption

Movie S2. Experiments of morphing wing controlled by a synstor circuit, a human operator, and an ANN in stall condition with an 18° angle of attack. A morphing wing in a wing tunnel is controlled by **a**, a synstor circuit, **b**, a human operator, and **c**, an ANN running in a computer. (Top) The videos show that the wing in a wing tunnel. (Bottom) Objective functions, $E = \frac{1}{2}\boldsymbol{s}^2$, with s_1 as the drag-to-lift force ratio and s_2 as the magnitude of the fluctuation of the drag-to-lift force ratio, is plotted against time t.

Sequential inference and reinforce learning executed in ANN

The sequential inference and reinforcement learning algorithms[46] executed in ANN is briefly described mathematically as following:

Initialize random synaptic weight matrix $\mathbf{W}(0)$ in ANN

Loop for episodes $n = 1, 2, \ldots, N_T$ till the morphing wing reaches the stable objective function E_e with $\dot{E} \approx 0$.

In the nth inference episode that lasted approximately 30 seconds, generate data $\boldsymbol{s}_L$, $\boldsymbol{y}$, and $\boldsymbol{W}(n)$ by following the inference function $f[y(t)|s_L(t), W(n)]$ in the ANN at discrete time steps $t = 0, 1, \ldots, N_D$ at the frequency of 12.5 KHz with input signals, $\boldsymbol{s}_L(t)$, as the deviations of the morphing wing position from the local target, $\boldsymbol{W}(n)$ as the ANN synaptic weight matrices in the nth inference episode, and $\boldsymbol{y}(t)$ as actuation pulses to control the morphing wing.

After the nth inference (flight) episode ends, start the nth learning episode.

In the nth learning episode, loop for discrete time steps $t = 0, 1, \ldots, N_D$, $\boldsymbol{\Gamma}(t+1) = \boldsymbol{\Gamma}(t) + r_L \mu^t (G(t) - b) \nabla_{\Gamma} \, ln[f[y(t)|\boldsymbol{s}_L(t), \boldsymbol{\Gamma}_k]]$, where $\boldsymbol{\Gamma}(0) = \boldsymbol{W}(n)$, learning rate $r_L > 0$, discount factor $\mu > 0$, $G(t) = \sum_{j=t+1}^{N_D} \mu^{j-t-1} R(j)$, baseline $b = \sum_t G(t) / N_D$, and reward $R(j)$.

Update $\boldsymbol{W}(n+1) = \boldsymbol{\Gamma}(N_D)$

The source codes are available on GitHub: https://github.com/Deo-Atharva/Morphing-Wing/tree/main](https://github.com/Deo-Atharva/Morphing-Wing/tree/main.
