# Supplementary material for: Super-Turing synaptic resistor circuits for intelligent morphing wing
Source: Commun Eng. 2025 Jun 16;4:109. doi: 10.1038/s44172-025-00437-y (PMC12170896; doi:10.1038/s44172-025-00437-y)
Supplement: Supplementary file 3 — Description of Additional Supplementary Files [file 44172_2025_437_MOESM3_ESM.pdf]

Description of Additional Supplementary Files

File name: Supplementary Movie 1

Description: Experiments of morphing wing controlled by a synstor circuit, a human operator, and an ANN in pre-stall condition with an 8° angle of attack. A morphing wing in a wing tunnel is controlled by a, a synstor circuit, b, a human operator, and c, an ANN running in a computer. (Top) The videos show that the wing in a wing tunnel. (Bottom) Objective functions, E=1/2 s^2, with s_1 as the drag-to-lift force ratio and s_2 as the magnitude of the fluctuation of the drag-to-lift force ratio, is plotted against time t.

File name: Supplementary Movie 2

Description: Experiments of morphing wing controlled by a synstor circuit, a human operator, and an ANN in stall condition with an 〖18〗^° angle of attack. A morphing wing in a wing tunnel is controlled by a, a synstor circuit, b, a human operator, and c, an ANN running in a computer. (Top) The videos show that the wing in a wing tunnel. (Bottom) Objective functions, E=1/2 s^2, with s_1 as the drag-to-lift force ratio and s_2 as the magnitude of the fluctuation of the drag-to-lift force ratio, is plotted against time t.
